# Supplementary material for: A surviving beta cell subpopulation enriched in patients with T1D
Source: bioRxiv. 2026 May 27:2026.05.15.725449. Preprint. [Version 2] doi: 10.64898/2026.05.15.725449 (PMC13228542; doi:10.64898/2026.05.15.725449)
Supplement: Supplement 2 [file NIHPP2026.05.15.725449v2-supplement-2.pdf]

bioRxiv preprint doi: https://doi.org/10.64898/2026.05.15.725449; this version posted May 27, 2026. The copyright holder for this preprint (which was not certified by peer review) is the author/funder, who has granted bioRxiv a license to display the preprint in perpetuity. It is made available under a CC-BY 4.0 International license.

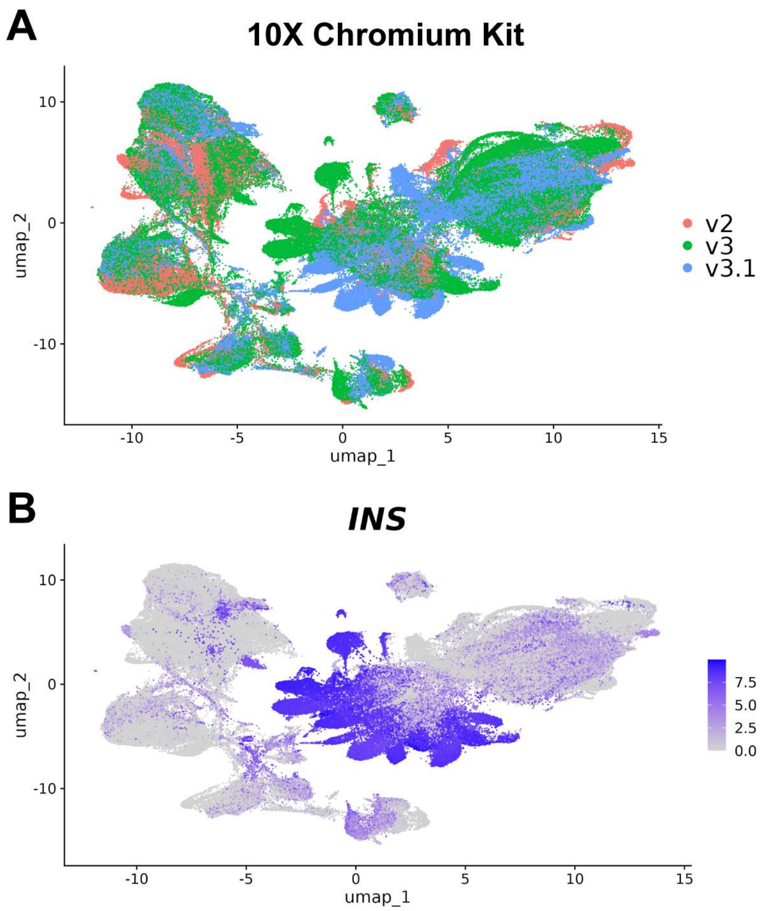

Supplementary Fig. 1: Technical differences in HPAP islet scRNAseq dataset
A: UMAP plot of HPAP islet cells after dimensionality reduction (PCA on normalized transcript counts) without integration methods. Cells are colored by 10X Chromium kit chemistry. B: Feature plot of *INS* expression to highlight beta cells.

bioRxiv preprint doi: https://doi.org/10.64898/2026.05.15.725449; this version posted May 27, 2026. The copyright holder for this preprint (which was not certified by peer review) is the author/funder, who has granted bioRxiv a license to display the preprint in perpetuity. It is made available under a CC-BY 4.0 International license.

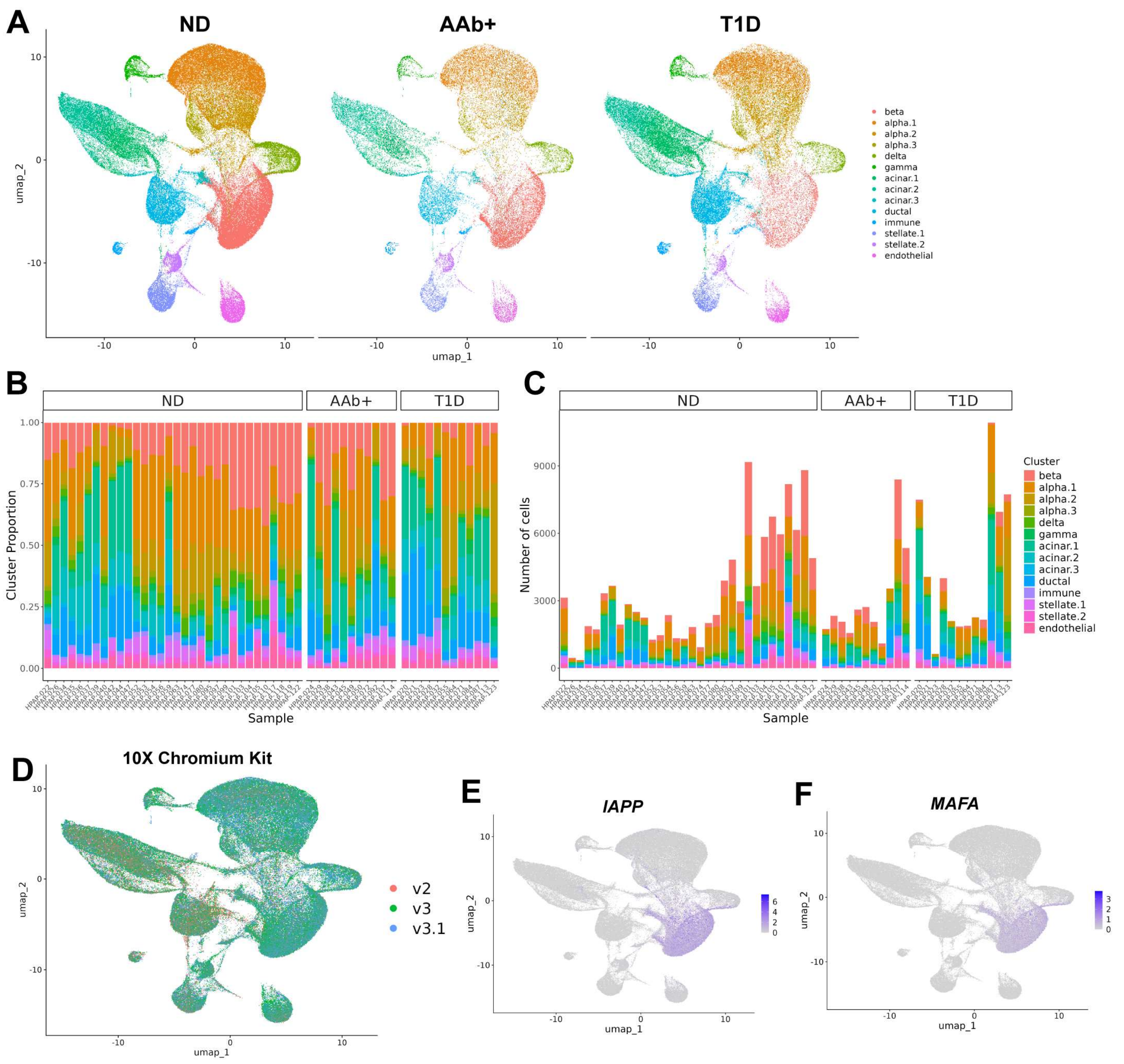

<u>Supplementary Fig. 2: Identification of beta cells in T1D, AAb+, and ND donors from HPAP scRNAseq data</u>

A: Integrated UMAP of HPAP islet cell types split by clinical status. B-C: Stacked bar plot showing frequency (B) and count (C) of each islet cell type per donor. Beta cell frequencies/counts shown here are those following post-islet clustering refinement and low feature count filtering of the beta cell population (Methods). D: Integrated UMAP of islet cell types by 10X Chromium reagent kit chemistry. E-F: Feature plots of islet expression of *IAPP* (E) and *MAFA* (F).

bioRxiv preprint doi: https://doi.org/10.64898/2026.05.15.725449; this version posted May 27, 2026. The copyright holder for this preprint (which was not certified by peer review) is the author/funder, who has granted bioRxiv a license to display the preprint in perpetuity. It is made available under a CC-BY 4.0 International license.

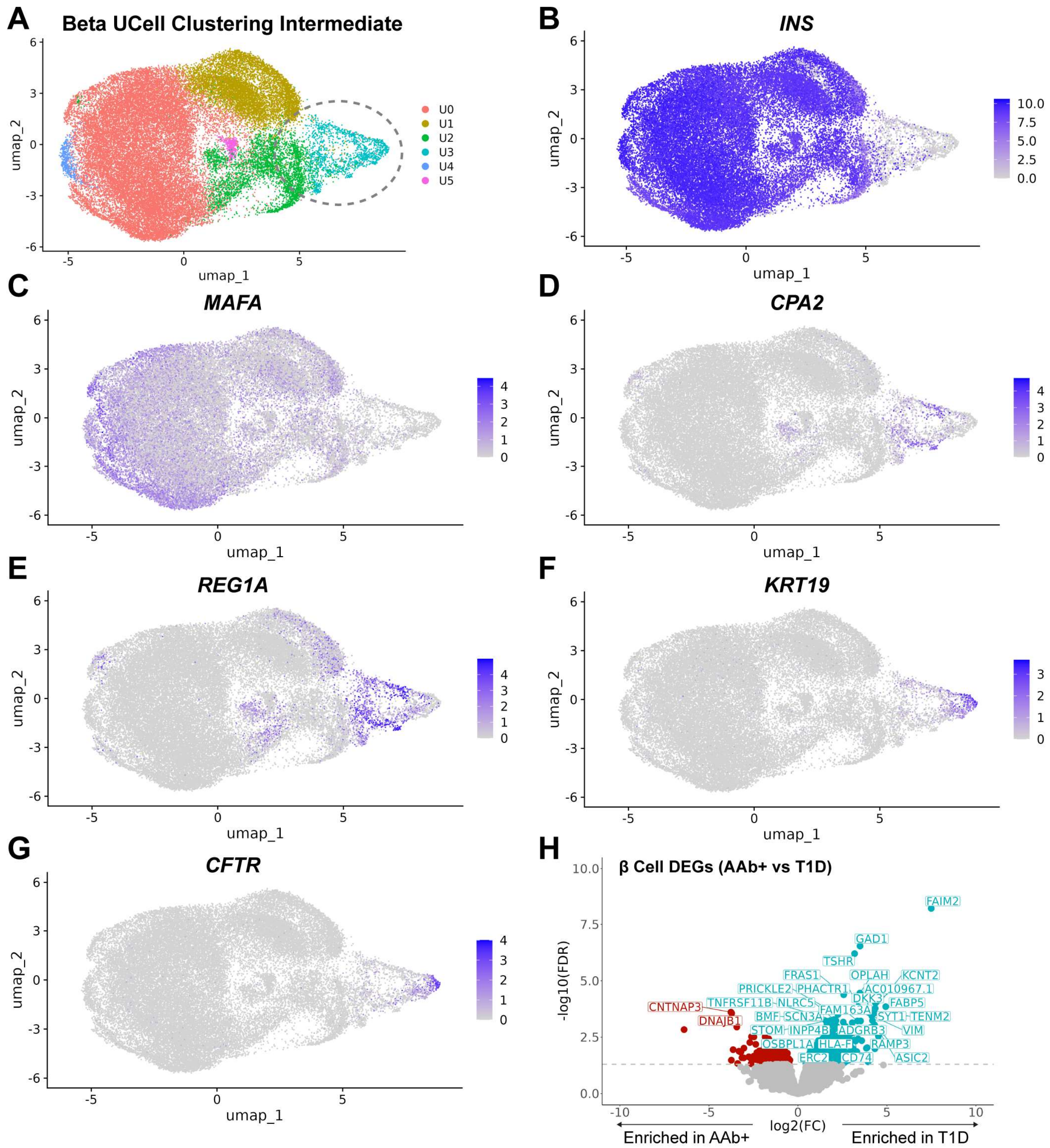

<u>Supplementary Fig. 3: Intermediate beta cell UCell clustering</u>

A: Intermediate beta cell clustering based on UCell scores (Methods). Gene sets for UCell score calculation are in Supplementary Table 3. Cells in cluster U3 (circled) were identified as likely contaminants and removed. B-G: Feature plots showing normalized expression of indicated transcripts. H: Volcano plot of DEGs between T1D and AAb+ beta cells calculated using edgeR with donor age, sex, BMI and 10X Chromium kit chemistry as covariates (as in Fig. 1F). A pseudobulk method was used by aggregating counts in beta cells by donor (Methods). BH-calculated FDR values are shown. The dashed line indicates FDR = 0.05.

bioRxiv preprint doi: https://doi.org/10.64898/2026.05.15.725449; this version posted May 27, 2026. The copyright holder for this preprint (which was not certified by peer review) is the author/funder, who has granted bioRxiv a license to display the preprint in perpetuity. It is made available under a CC-BY 4.0 International license.

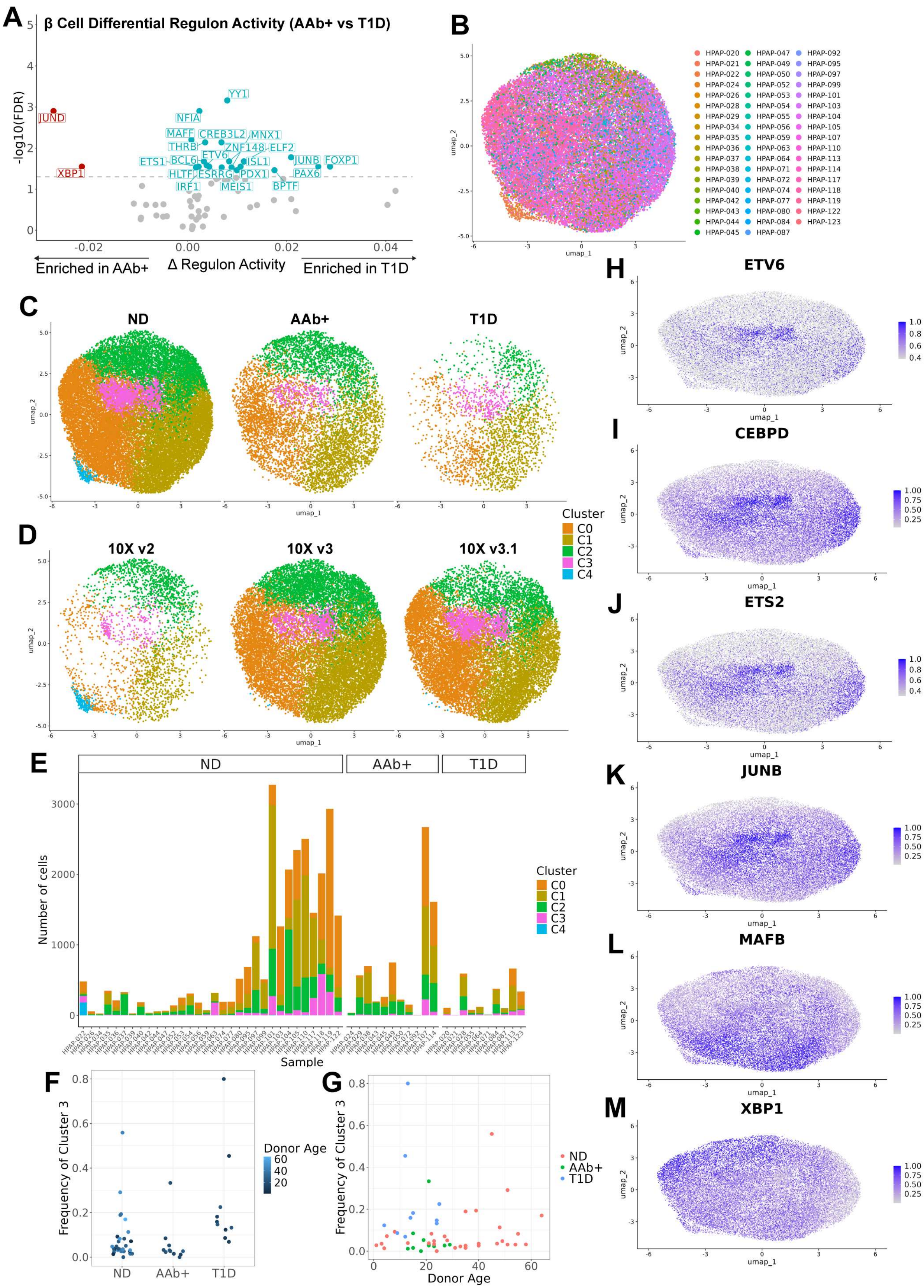

bioRxiv preprint doi: https://doi.org/10.64898/2026.05.15.725449; this version posted May 27, 2026. The copyright holder for this preprint (which was not certified by peer review) is the author/funder, who has granted bioRxiv a license to display the preprint in perpetuity. It is made available under a CC-BY 4.0 International license.

Supplementary Fig. 4: Identification of a T1D enriched beta cell population through GRN inference-based clustering

A: Volcano plot of differentially active regulons between T1D and AAb+ beta cells (as in Fig. 2A). Regulon scores were averaged by donor. Differential activity testing was performed using a linear model with donor age, sex, BMI and 10X Chromium kit chemistry as covariates. BH-calculated FDR values are shown. The dashed line indicates FDR = 0.05. B: Beta cell UMAP by individual donor. C-D: Beta cell UMAP split by clinical status (C) and 10X Chromium reagent kit chemistry (D). E: Count of each beta cell cluster per donor. F: Frequency of C3 beta cells (among all beta cells) by clinical status. Each dot is one donor, shaded by donor age. G: Frequency of C3 beta cells (among all beta cells) versus donor age. Each dot is one donor, colored by clinical status. H-M: Feature plots of rank-normalized regulon activity scores.

bioRxiv preprint doi: https://doi.org/10.64898/2026.05.15.725449; this version posted May 27, 2026. The copyright holder for this preprint (which was not certified by peer review) is the author/funder, who has granted bioRxiv a license to display the preprint in perpetuity. It is made available under a CC-BY 4.0 International license.

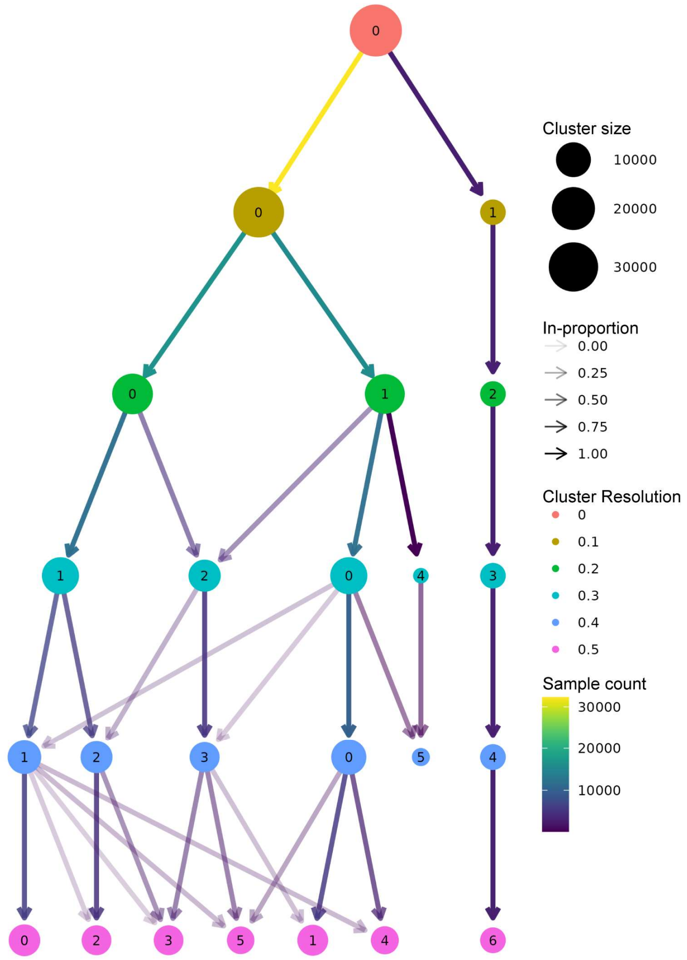

Supplementary Fig. 5: Beta cell cluster 3 is stable across clustering resolutions

Clustree plot showing clustering solutions at different resolutions (Zappia & Oshlack, 2018). A resolution of 0.3 was used for the UMAP in Fig. 2B. Edges from parent nodes contributing less than 10% of cells to the child node are not shown.

bioRxiv preprint doi: https://doi.org/10.64898/2026.05.15.725449; this version posted May 27, 2026. The copyright holder for this preprint (which was not certified by peer review) is the author/funder, who has granted bioRxiv a license to display the preprint in perpetuity. It is made available under a CC-BY 4.0 International license.

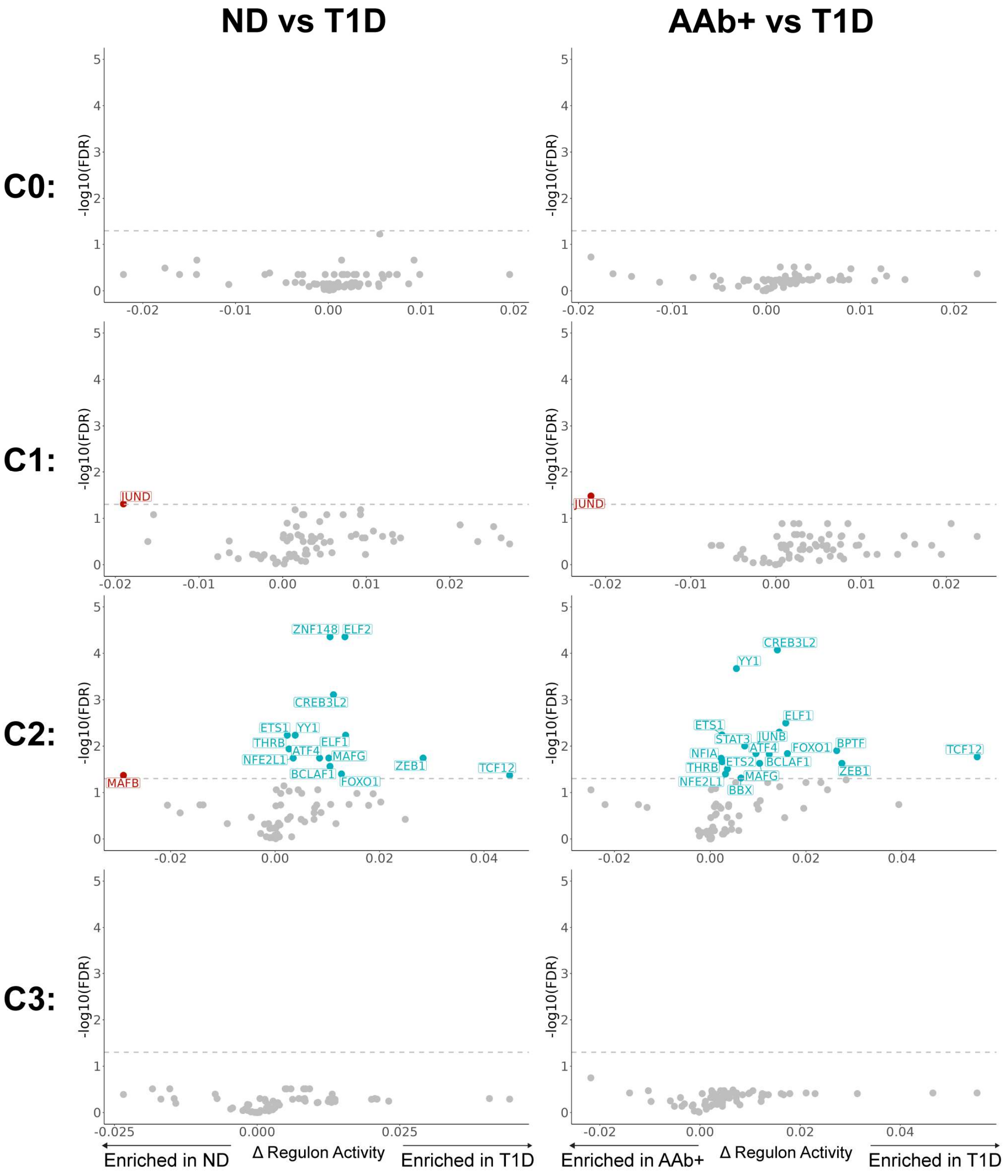

<u>Supplementary Fig. 6: Differential regulon activity between T1D and ND/AAb+ beta cells within each beta cell subcluster</u>

Volcano plots of differentially active regulons between T1D and ND/AAb+ beta cells for each beta cell cluster. Regulon scores were averaged by donor for each cluster. Donors with less than 5 beta cells in a particular cluster were excluded for comparisons within that cluster. Differential activity testing was performed using a linear model with donor age, sex, BMI and 10X Chromium kit chemistry as covariates. BH-calculated FDR values are shown on the y axis. Dashed lines indicate FDR = 0.05.

bioRxiv preprint doi: https://doi.org/10.64898/2026.05.15.725449; this version posted May 27, 2026. The copyright holder for this preprint (which was not certified by peer review) is the author/funder, who has granted bioRxiv a license to display the preprint in perpetuity. It is made available under a CC-BY 4.0 International license.

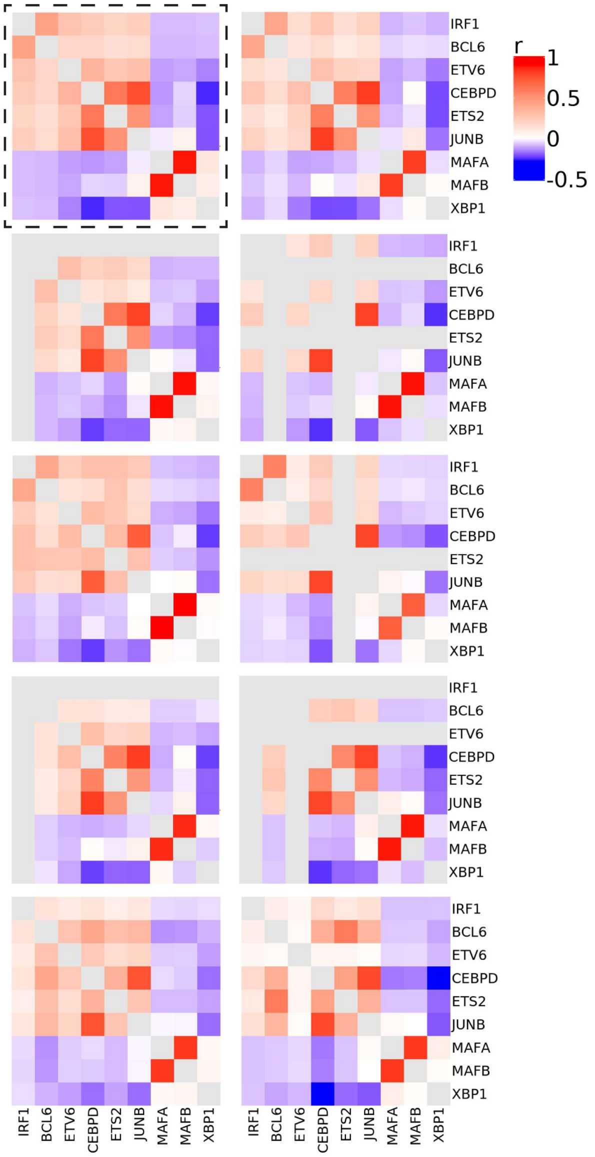

Supplementary Fig. 7: Stability of RegDiffusion model for SCENIC GRN inference

Regulons were scored after independently re-training the RegDiffusion model 9 additional times on the same beta cell population as used for the original run (original run used for all analyses in the dashed box, 10 total trainings). Pairwise Pearson correlation matrices for beta cell activity of the top 6 up and top 3 down C3-enriched regulons are shown. Greyed rows/columns indicate that the regulon was not detected in a given run. The matrix diagonal is also colored grey.

bioRxiv preprint doi: https://doi.org/10.64898/2026.05.15.725449; this version posted May 27, 2026. The copyright holder for this preprint (which was not certified by peer review) is the author/funder, who has granted bioRxiv a license to display the preprint in perpetuity. It is made available under a CC-BY 4.0 International license.

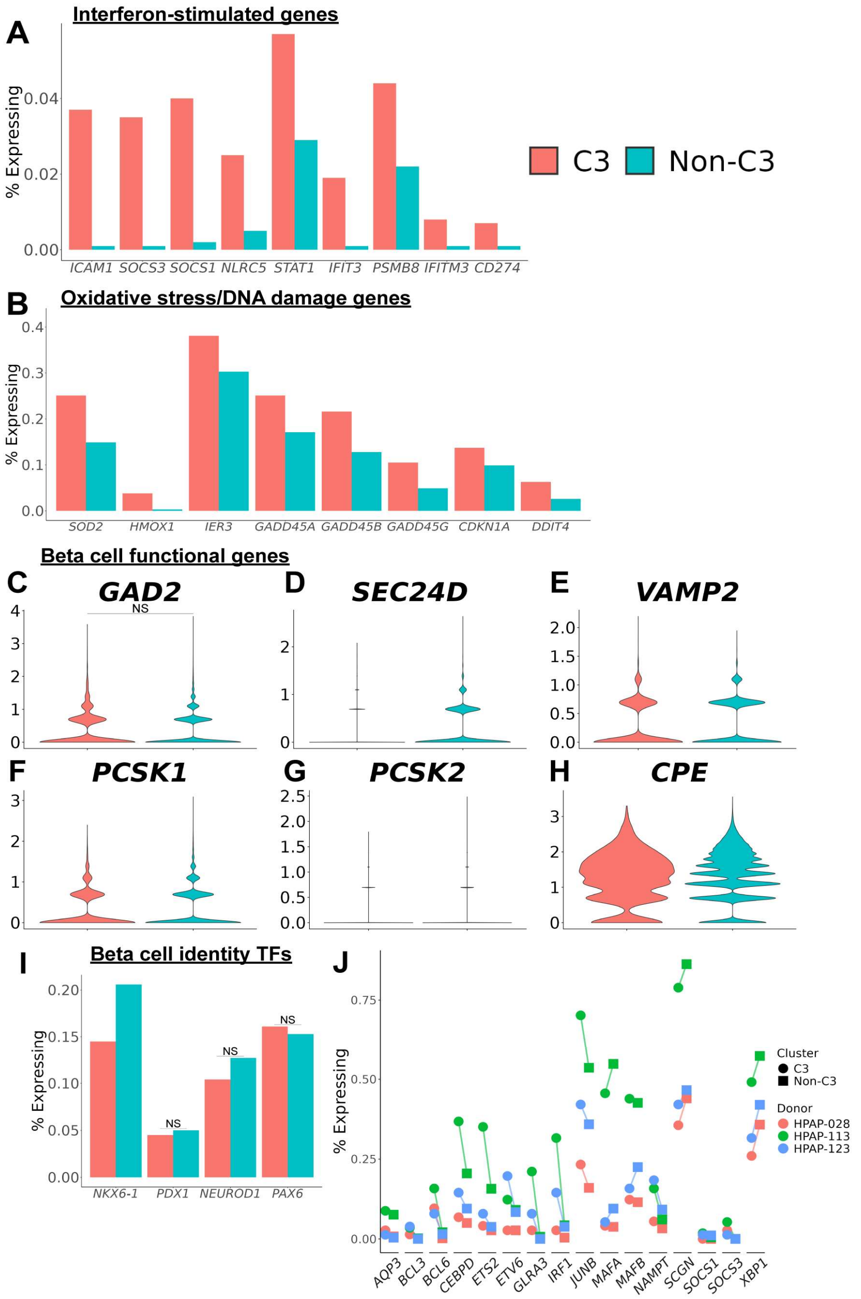

Supplementary Fig. 8: Transcriptional profile of cluster 3 beta cells

A: Frequency of beta cells expressing indicated ISGs between C3 and non-C3 beta cells. B: Frequency of beta cells expressing indicated oxidative stress/DNA damage genes between C3 and non-C3 beta cells. C-H: Violin plots showing expression of indicated transcripts for C3 and non-C3 beta cells. I: Frequency of beta cells expressing lineage-defining beta cell TFs between C3 and non-C3 beta cells. For all panels, all adjusted p values (Bonferroni) < 0.05 unless indicated by NS (not significant). P values derived from DEG testing (Methods). Full cluster 3 differential expression tabular data is available in Supplementary Table 9. J: Percent of cells expressing indicated transcripts for the 3 T1D samples with the most C3 beta cells. Trends are shown (not significance) due to low cell counts in individual donors.

bioRxiv preprint doi: https://doi.org/10.64898/2026.05.15.725449; this version posted May 27, 2026. The copyright holder for this preprint (which was not certified by peer review) is the author/funder, who has granted bioRxiv a license to display the preprint in perpetuity. It is made available under a CC-BY 4.0 International license.

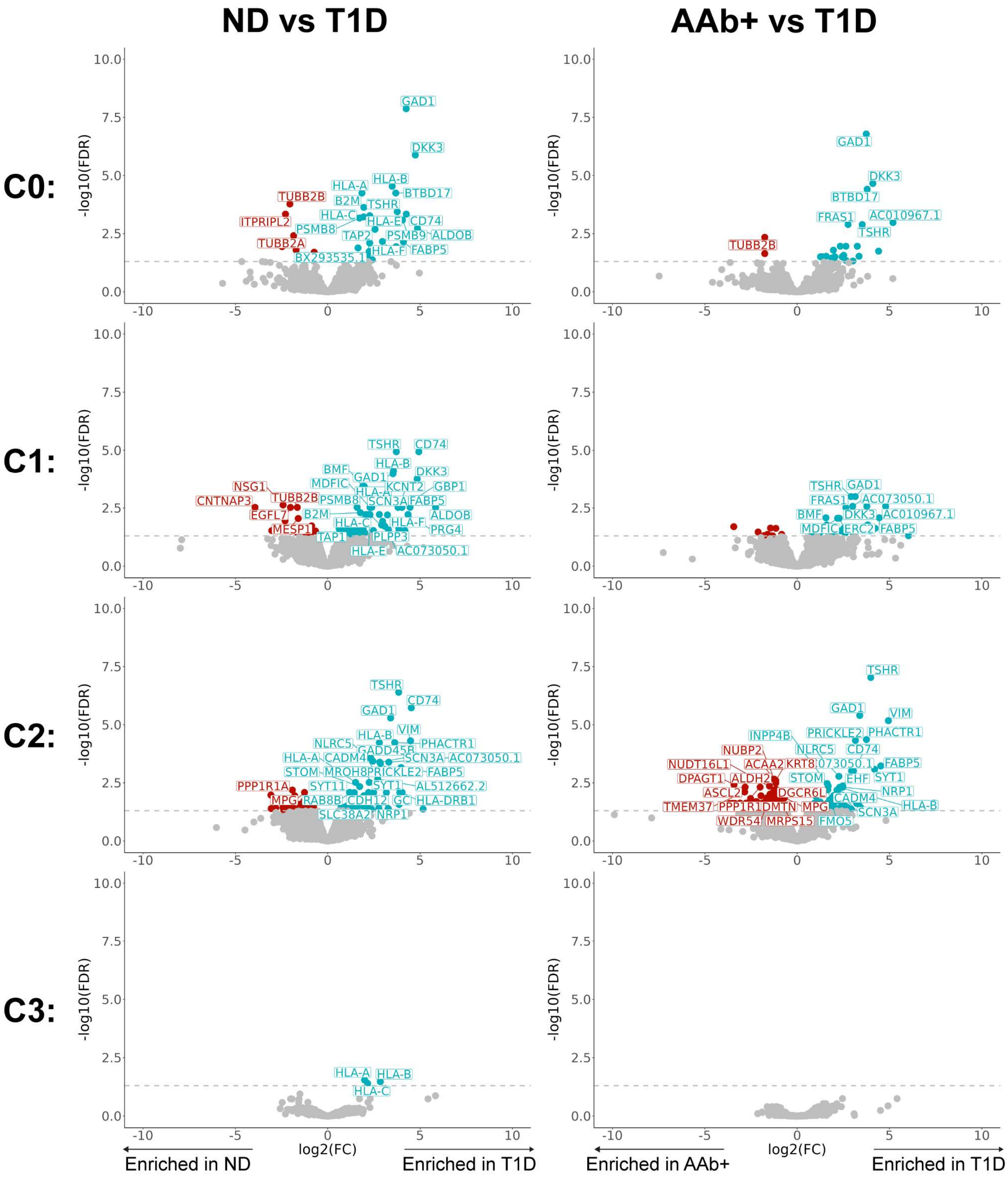

<u>Supplementary Fig. 9: DEGs between T1D and ND/AAb+ beta cells within each beta cell subcluster</u>

Volcano plots of DEGs between T1D and ND/AAb+ beta cells within each cluster calculated using edgeR with donor age, sex, BMI and 10X Chromium kit chemistry as covariates. A pseudobulk method was used by aggregating counts by donor. Donors with less than 5 beta cells in a particular cluster were excluded for comparisons within that cluster. BH-calculated FDR values are shown on the y axis. Dashed lines indicate FDR = 0.05.

bioRxiv preprint doi: https://doi.org/10.64898/2026.05.15.725449; this version posted May 27, 2026. The copyright holder for this preprint (which was not certified by peer review) is the author/funder, who has granted bioRxiv a license to display the preprint in perpetuity. It is made available under a CC-BY 4.0 International license.

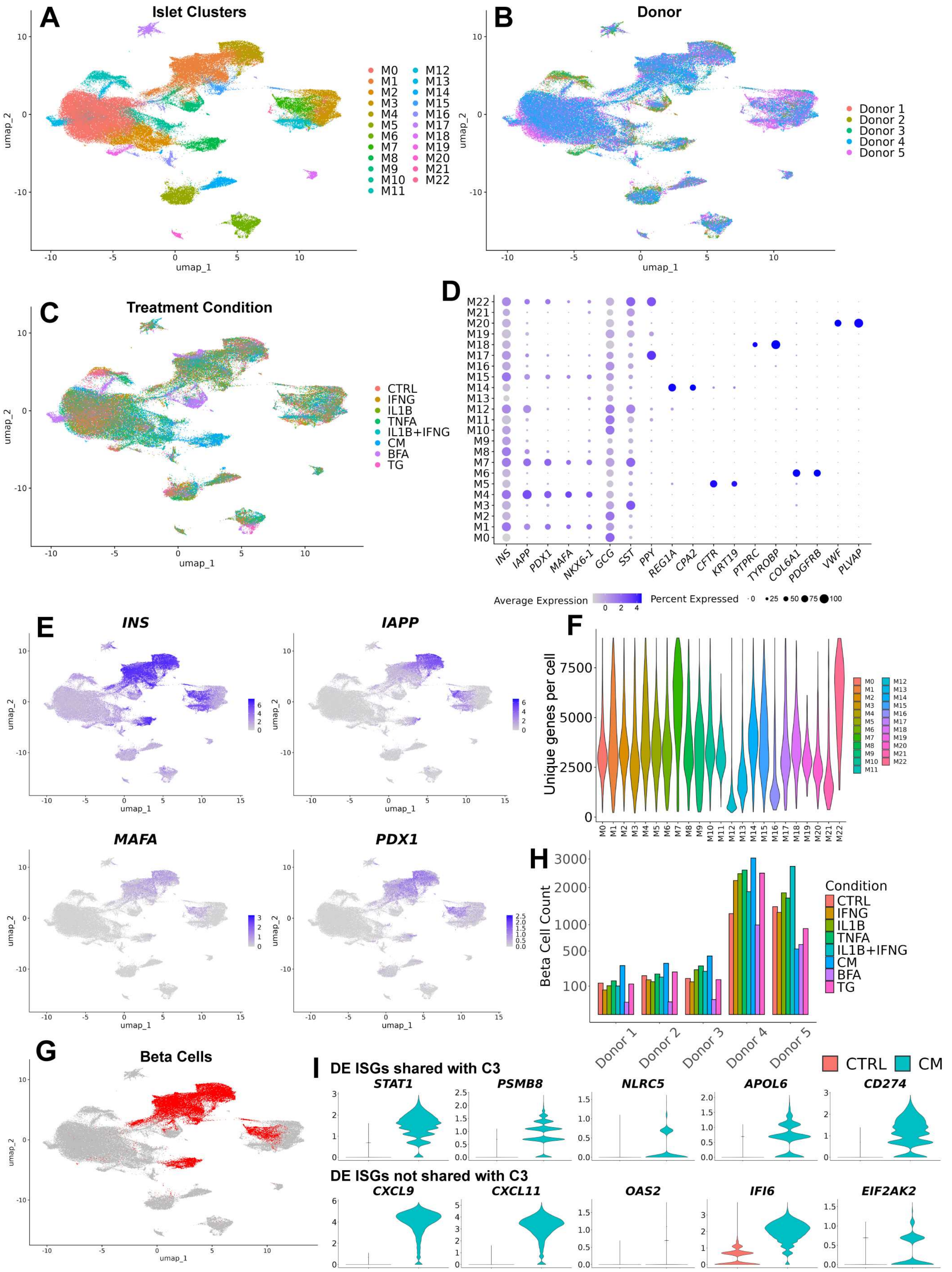

bioRxiv preprint doi: https://doi.org/10.64898/2026.05.15.725449; this version posted May 27, 2026. The copyright holder for this preprint (which was not certified by peer review) is the author/funder, who has granted bioRxiv a license to display the preprint in perpetuity. It is made available under a CC-BY 4.0 International license.

Supplementary Fig. 10: Beta cell identification and gene expression from Maestas et al. in vitro scRNAseq data

A: Integrated UMAP from reanalysis of in vitro islet scRNAseq data Maestas et al. B-C: UMAP showing cells colored by donor (B) and treatment condition (C; CTRL: control, CM: cytokine mix with IFNG+IL1B+TNFA, BFA: Brefeldin A, TG: Thapsigargin). D: Dot plot of scaled islet marker gene expression within each cluster. E: Feature plots of beta cell marker transcripts. F: The number of unique genes expressed per cell within each cluster. G: UMAP showing cells identified as beta cells from Maestas et al. scRNAseq data (highlighted in red, beta cells defined as cells belonging to clusters M1, M4, M7, M8, or M15). H: Beta cell counts recovered for each sample (donor + treatment combination). I: Violin plots of in vitro cytokine-treated and control beta cells (pooled from all donors). All panels are differentially expressed with adjusted p values (Bonferroni) < 0.05. Shared ISGs are those differentially expressed in both in vivo C3 vs non-C3 beta cells (HPAP) and in vitro cytokine-treated vs control beta cells (Maestas et al.). Non-shared ISGs are those only differentially expressed by in vitro cytokine-treated vs control beta cells (Maestas et al.). Full tabular data for HPAP C3 DEGs are found in Supplementary Table 9 and for Maestas et al. in Supplementary Table 13.

bioRxiv preprint doi: https://doi.org/10.64898/2026.05.15.725449; this version posted May 27, 2026. The copyright holder for this preprint (which was not certified by peer review) is the author/funder, who has granted bioRxiv a license to display the preprint in perpetuity. It is made available under a CC-BY 4.0 International license.

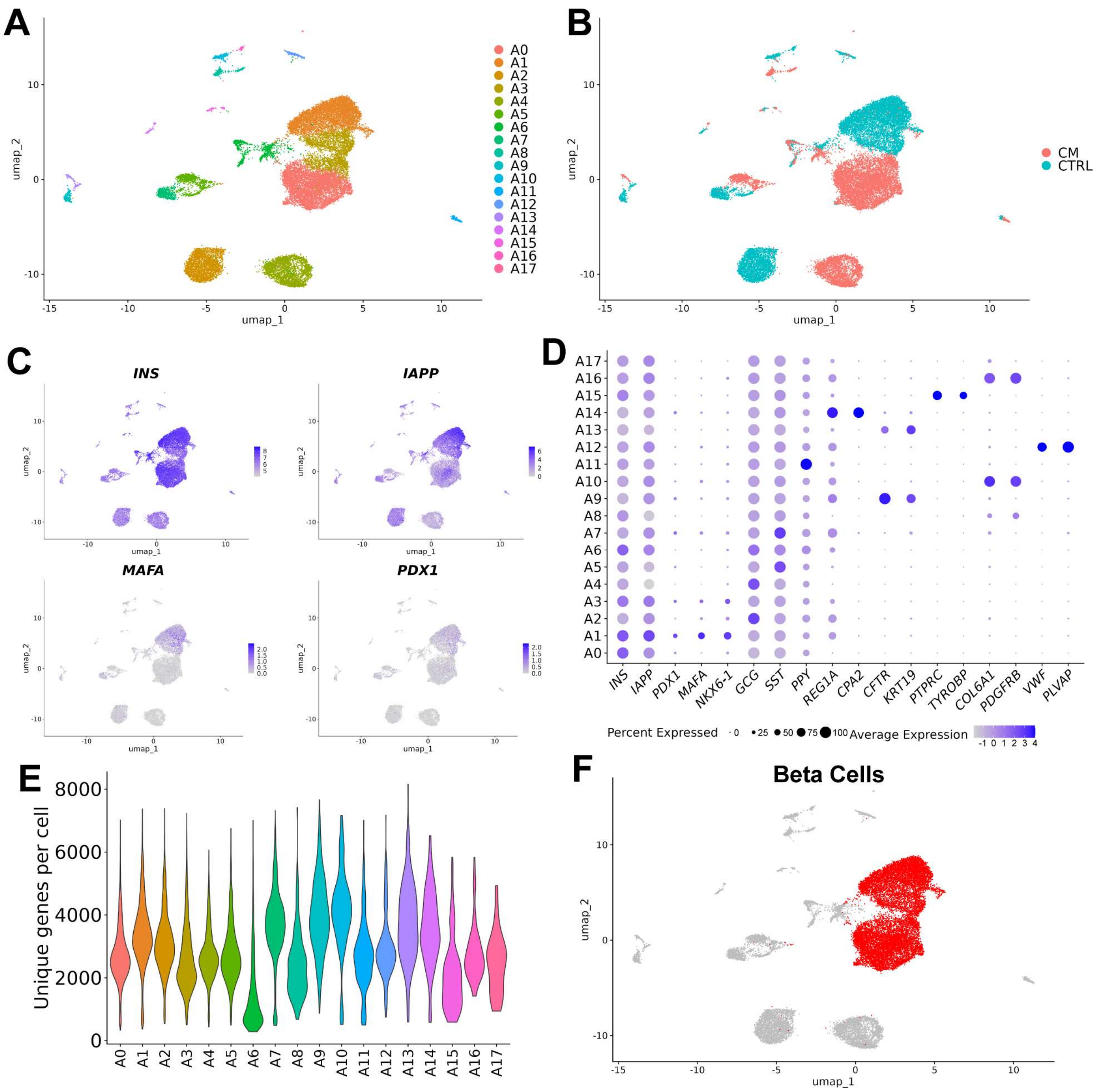

Supplementary Fig. 11: Beta cell identification from Maestas et al. in vitro snMultiome data

A: Integrated ATAC and RNA UMAP from Maestas et al. islet snMultiome data. B: UMAP showing cells colored by treatment condition (CTRL: control, CM: cytokine mix with IFNG+IL1B+TNFA). C: Feature plots of beta cell marker transcripts. D: Dot plot of scaled islet marker gene expression within each cluster. E: The number of unique genes expressed per cell within each cluster. F: UMAP showing cells identified as beta cells from Maestas et al. snMultiome data (highlighted in red, beta cells defined as cells belonging to clusters A0, A1, or A3).

bioRxiv preprint doi: https://doi.org/10.64898/2026.05.15.725449; this version posted May 27, 2026. The copyright holder for this preprint (which was not certified by peer review) is the author/funder, who has granted bioRxiv a license to display the preprint in perpetuity. It is made available under a CC-BY 4.0 International license.

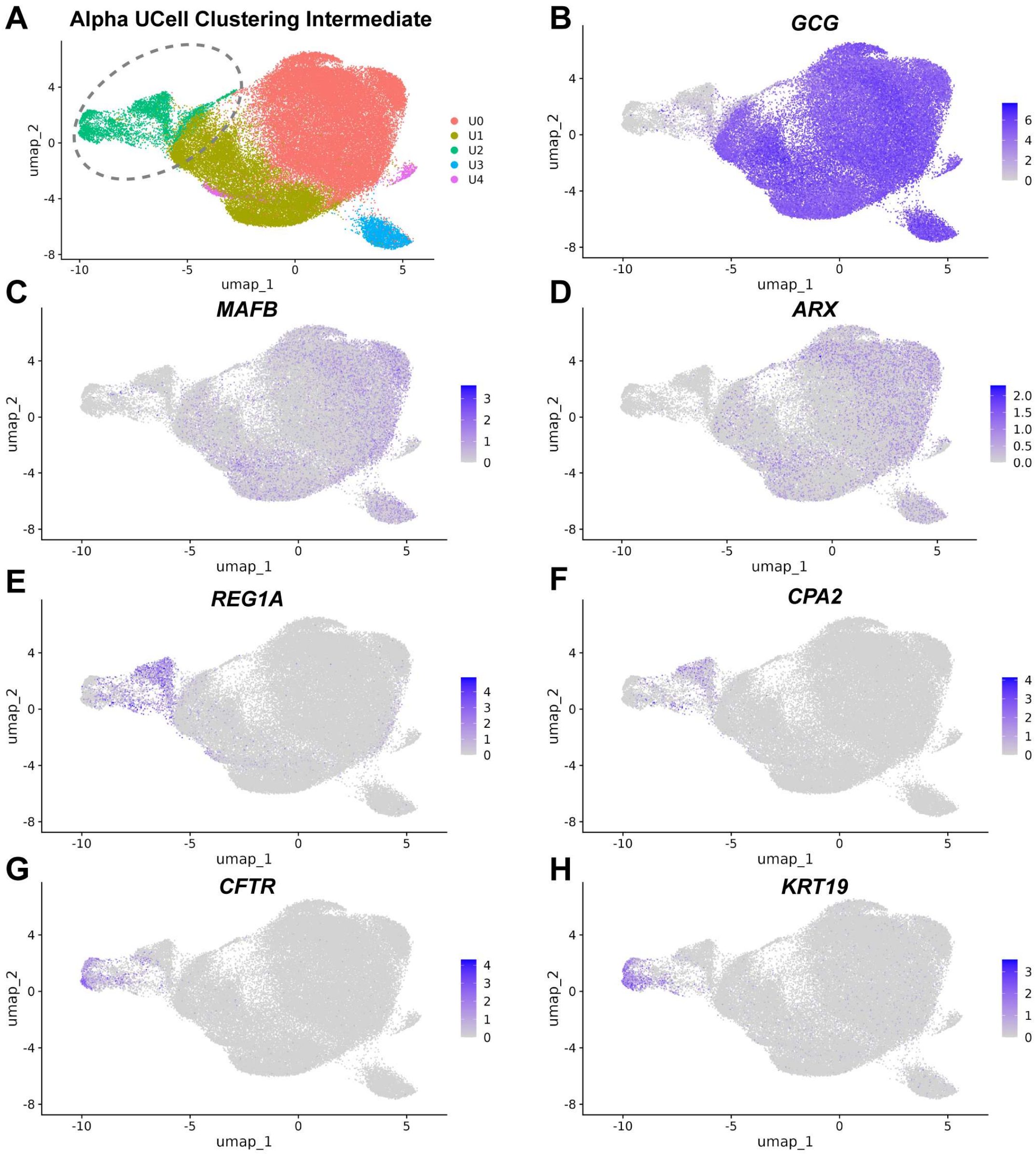

Supplementary Fig. 12: Intermediate alpha cell UCell clustering

A: Intermediate alpha cell clustering based on UCell scores (Methods). Gene sets for UCell score calculation are in Supplementary Table 3. Cells in cluster U2 (circled) were identified as likely contaminants and removed. B-H: Feature plots showing normalized expression of indicated transcripts.

bioRxiv preprint doi: https://doi.org/10.64898/2026.05.15.725449; this version posted May 27, 2026. The copyright holder for this preprint (which was not certified by peer review) is the author/funder, who has granted bioRxiv a license to display the preprint in perpetuity. It is made available under a CC-BY 4.0 International license.

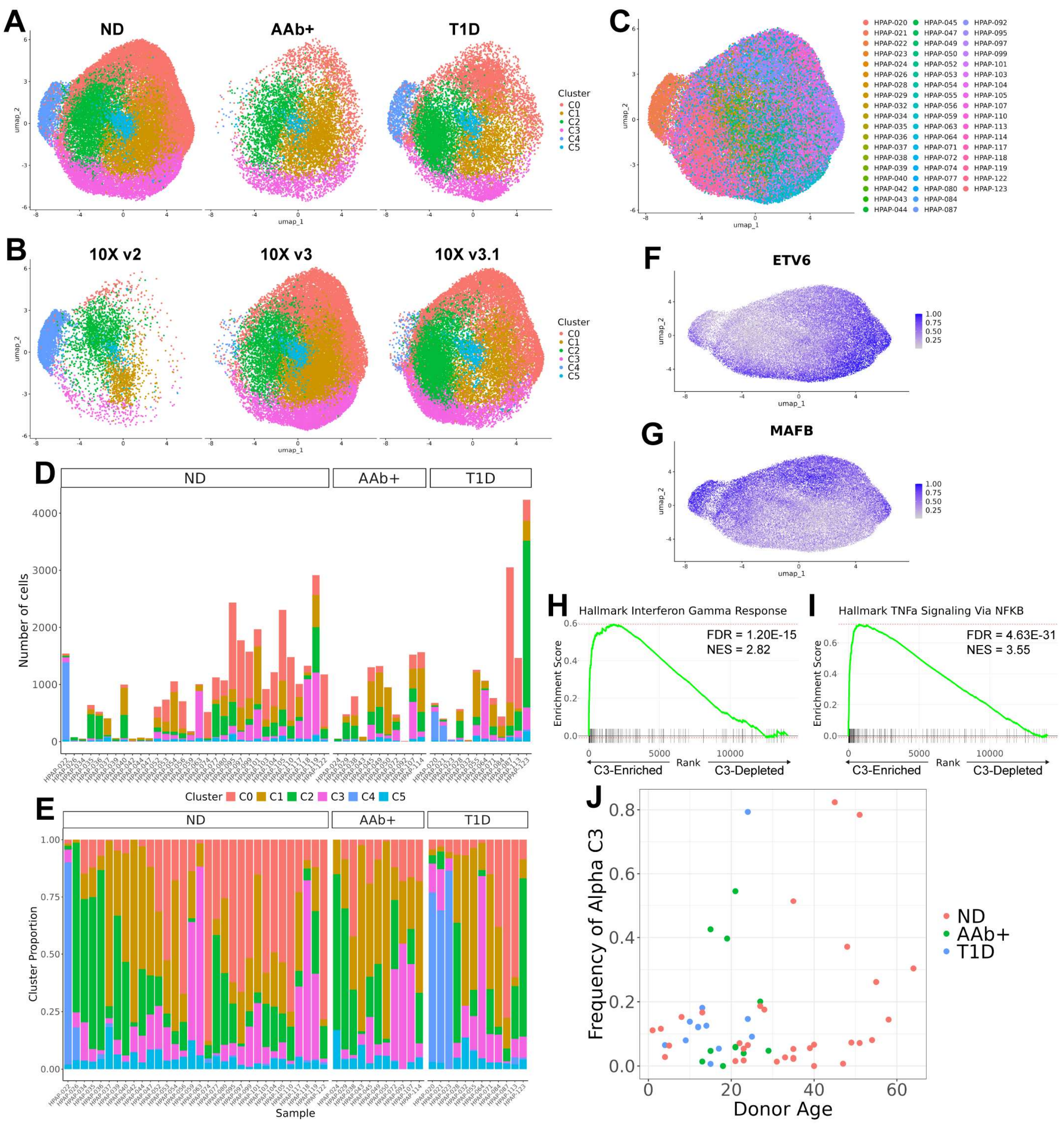

<u>Supplementary Fig. 13: Identification of an alpha cell population homologous to cluster 3 beta cells</u>

A-B: Alpha cell UMAP split by clinical status (A) and 10X Chromium reagent kit chemistry (B). C: Alpha cell UMAP by individual donor. D: Count of each alpha cell cluster per donor. E: Frequency of each alpha cell cluster per donor. F-G: Feature plots of rank-normalized regulon activity scores. H-I: Gene set enrichment plots for selected pathways (full list of gene sets in Supplementary Table 3). Genes were ranked by fold change in C3 alpha cells (vs non-C3 alpha cells), with filtering of lowly expressed genes (Methods, full table of GSEA output in Supplementary Table 20). BH-calculated FDR values are shown. NES: normalized enrichment score. J: Frequency of C3 alpha cells (among all alpha cells) versus donor age. Each dot is one donor, colored by clinical status.
